# Progressive changes in descriptive discourse in First Episode Schizophrenia: a longitudinal computational semantics study

**DOI:** 10.1038/s41537-022-00246-8

**Published:** 2022-04-12

**Authors:** Maria Francisca Alonso-Sánchez, Sabrina D. Ford, Michael MacKinley, Angélica Silva, Roberto Limongi, Lena Palaniyappan

**Affiliations:** 1 CIDCL, Fonoaudiología, Facultad de Medicina, Universidad de Valparaíso, Valparaíso, Chile; 2 Robarts Research Institute, Western University, London, ON Canada; 3 Lawson Health Research Institute, London, ON Canada; 4 Douglas Mental Health University Institute , McGill University, Montreal, QC Canada; 5 Department of Psychiatry, Schulich School of Medicine and Dentistry, Western University, London, ON Canada

**Keywords:** Human behaviour, Schizophrenia

## Abstract

Computational semantics, a branch of computational linguistics, involves automated meaning analysis that relies on how words occur together in natural language. This offers a promising tool to study schizophrenia. At present, we do not know if these word-level choices in speech are sensitive to the illness stage (i.e., acute untreated vs. stable established state), track cognitive deficits in major domains (e.g., cognitive control, processing speed) or relate to established dimensions of formal thought disorder. In this study, we collected samples of descriptive discourse in patients experiencing an untreated first episode of schizophrenia and healthy control subjects (246 samples of 1-minute speech; *n* = 82, FES = 46, HC = 36) and used a co-occurrence based vector embedding of words to quantify semantic similarity in speech. We obtained six-month follow-up data in a subsample (99 speech samples, *n* = 33, FES = 20, HC = 13). At baseline, semantic similarity was evidently higher in patients compared to healthy individuals, especially when social functioning was impaired; but this was not related to the severity of clinically ascertained thought disorder in patients. Across the study sample, higher semantic similarity at baseline was related to poorer Stroop performance and processing speed. Over time, while semantic similarity was stable in healthy subjects, it increased in patients, especially when they had an increasing burden of negative symptoms. Disruptions in word-level choices made by patients with schizophrenia during short 1-min descriptions are sensitive to interindividual differences in cognitive and social functioning at first presentation and persist over the early course of the illness.

## Introduction

Language disorganization is a prominent feature in psychosis, and it is commonly observed initially as a disorder in generating interpersonal discourse. This produces a significant functional impairment for the patient as it interferes with one’s ability to describe or explain attributes and thus socialize in everyday life^1^. When engaged in a descriptive discourse of a concrete referent, such as a picture, to a second person, patients with schizophrenia make unusual word choices^2^, exhibit repetitiveness and convey less information (referred to as ‘weakening of goal’^3^ or ‘poverty of content’^4^) than healthy controls^3,5^. In particular, the restricted repertoire of word selection, characterized by smaller loops of word-to-word connectivity that occurs with more proximal repeats in selected words, becomes apparent even before overt psychosis^6^, predicts later onset of psychosis^6,7^, becomes more pronounced during the first episode^7^, and relates to reduced social and occupational functioning^8^.

Descriptive discourse involves multiple levels of cognitive processing^9^ to integrate parts and attributes of the whole to produce a descriptive schema^10^. We often employ descriptions in the service of rhetorical functions (i.e., ways to inform, argue, persuade someone) through our choice of words. In psycholinguistic terms, descriptive discourse requires semantic competence^1^ and appropriate lexical access to a connectionist system of words organized by their conceptual relationships with one another^10^. In this context, lexical units (words) with a higher likelihood of occurring together have a stronger connection or a smaller distance between them (distributional semantics)^11^. This idea follows the original spreading-activation hypothesis of lexical representations in the brain^12^. Competitive theories of lexical selection assume that lexical representations must overcome interference from the neighbour’s activation through lateral inhibition^13^. Applying this to the picture description task, a failure of appropriate selection via inhibition at the lexical level may give rise to a description that is replete with words that are highly associated with each other, without capturing the different attributes of the picture at hand.

A proactive ‘top-down’ contextual guidance during discourse can reduce the overreliance on the bottom-up activation of the lexico-semantic network for word selection^14^. A breakdown in this contextual guidance, implemented as top-down inhibition from inferior frontal to semantic storage systems^15^, has been variously described in schizophrenia^16^. A large body of literature demonstrates frontal cognitive control deficits in schizophrenia, exemplified by reduced performance in the colour-word Stroop Task that tests one’s ability to inhibit competing semantic categorical representations when choosing a word^17^. In particular, the increased Stroop interference effect, in both response time and accuracy measures, has been interpreted as a marker of impaired inhibitory feature of cognitive control^17^. Abnormalities in this aspect of cognitive control have been previously related to conceptual disorganization^18^, a symptom related to linguistic aberrations in schizophrenia^19,20^. In addition, inter-individual variations in processing speed also influences lexical access^21^. In fact, reduced processing speed is the neurocognitive domain with the strongest correlation with disorganisation^22,23^. On this basis, we can expect deficits in cognitive control and processing speed to influence word selection during a descriptive discourse in patients with schizophrenia.

When examining similarity among the words used during discourse, there are broadly 2 approaches. One approach is to count the instances of repetition of a word. This phenomenon is described as perseveration in clinical rating scales^3,4^. A measure of lexical diversity called Type-Token Ratio (TTR; the ratio of unique to total words in a text) is computed based on such repetitions. As exact repetitions are relatively rare, perseveration is often not detectable in cross-sectional interviews^24,25^, and results from TTR studies are inconclusive^22–25^ with more recent studies showing both increased^26^ and reduced^27,28^ TTR in schizophrenia. Graph theoretical approaches that rely on the proximity between two repetitions, rather than counting the instances of repetitions, appear to carry more diagnostic and prognostic information in schizophrenia^8,29,30^. However, this approach cannot distinguish meaningful repetitions of informational value (e.g., “He liked the idea of travel, and the memory of travel, but not travel itself” [― Julian Barnes, Flaubert’s *Parrot*]) from the problematic repetitions that affect communication. The second approach is to employ distributional semantics to estimate the similarity, rather than exact repetition, among a set of words. This taps on a network-based distributional model of words. If lexical units are interconnected based on their co-occurrence in everyday language, then similarity among a set of words used during a discourse can be quantified based on this distributional co-occurrence.

Approaches from distributional semantics have been applied to study the relationship among words produced during various speech elicitation tasks in schizophrenia. The most popular approach, introduced by Elvevåg^31^, involves the use of latent semantic analysis (LSA) that taps on the document-level statistical co-occurrence of words in a large corpus of written texts; this determines their position in the semantic space based on the “company they keep”. The cosine similarity of this spatial index can then be computed among the words spoken by a patient. Several studies have demonstrated the potential utility of distributional semantics in predicting the onset of psychosis^2,32,33^, examining thought disorder^34–36^ and its neuroanatomical basis of linguistic disruptions in psychosis^37^. Other similar methods improved on LSA, by weighting the statistics of co-occurrence based on the actual proximity of words in the sentences occurring in the reference corpora^38–44^. We employ one such improved method (CoVec), that has been used previously in the study of semantic fluency tasks in schizophrenia^45,46^.

Cosine similarity can be computed between words that are adjacent to each other within a frame, indicating if words proximal to each other are sampled from a narrow semantic space^43–46^. Cosine similarity among the full frame of words in a descriptive text (termed Mean Similarity in CoVec) indicates the semantic diversity of all words employed to provide the complete description of a referent. As spoken text rarely assumes the form of sentences, a finite moving window (e.g., 5, 10 or 20 words size^45–48^) is also used to define frames of measurement. In our case, the full 1-minute description of a picture constitutes the frame of interest (ASW-F or Average Similarity of Words in Full Frame) to define semantic similarity, with the average similarity estimated from a 10-word moving window (ASW-10) as a secondary measure.

Studies employing distributional semantics have often used the term coherence to describe the degree of similarity (e.g. local coherence^4^, semantic coherence^31^, or cohesion^49^) or incoherence when describing its pathological reduction^34,44^ (see^38,50^ for a review). While several NLP studies have employed the term coherence in this sense, we use the term ‘similarity’ rather than coherence when employing cosine similarity. Hoffman pointed out that coherence is a psychological experience of a listener and not a property of a text^51^. To experience a text as coherent, the listener must employ a subjective interpretive synthesis that depends on their experience of the referent (i.e., drawing the linkage between the described object and the presented text) and directionality (i.e. which word or idea came first), in addition to the dependency among the lexical/semantic units. Furthermore, words with a low probability of co-occurrence can be coherently juxtaposed in certain contexts, that may not be apparent from the text itself. Also, metadiscursive (frameshifting^51^) elements can improve coherence for a listener (e.g., changing topics by saying “to go on a tangent for a bit”). For these reasons, we do not infer semantic *coherence* but only *similarity* from the indices of distributional semantics employed here.

We hypothesize that when faced with the task of describing an unfamiliar concrete referent^52^ (a picture), patients with schizophrenia will employ words with a higher probability of semantic co-occurrence. We expect abnormal semantic similarity to be evident in the untreated, first episode phase of illness and relate to formal thought disorder, reduced cognitive control and processing speed in patients. To test if the abnormality in semantic similarity was specific to the picture description task, wherein the word choices we make depend on the descriptive nature of discourse, we studied similarity of word choices in a conventional category fluency task. We will also address several confounds such as years of education^53^, migrant status, parental socioeconomic status, bilingualism^54^ and antipsychotic use (especially those with high occupancy of dopamine D2 receptors)^55^ that are critical for the current study as they typically influence schizophrenia prognosis^56^.

Several previous cross-sectional studies have related language and communication difficulties to social functioning among patients^57,58^. Interestingly, studies investigating longitudinal changes of language remains scarce in psychosis^59^, even though worsening of formal thought disorder over time has been shown to relate to progressive worsening of social and occupational outcome^60^. Furthermore, exposure to antipsychotics, that occurs when treatment is initiated in FES, is also associated with worsening of speech measures, especially word selection measures^55^. We anticipate that, unlike healthy controls who will show no changes in their word-level choices over the time, a persistent or worsening deficit in semantic similarity over time will be seen among FES patients.

To this end, we recruited a sample of acutely unwell, first-episode patients with < 14 days of lifetime exposure to antipsychotics at baseline. These patients were then treated in an early intervention clinic and followed up after 6 months to examine their discourse stability. This allowed us to relate treatment variables (antipsychotic exposure) as well as outcome variables (SOFAS scores) to word similarity measures over time.

## Results

### Demographic and clinical characteristics

Healthy controls and the FES group (First Episode Schizophrenia) did not significantly differ in age, gender distribution or educational level. In the FES group, 20% of the participants were first-generation immigrants (determined from self-report) while 30% of the matched HC group were first-generation immigrants. There was no group difference in the use of English as the first language (82% FES and 88% HC had English as the first language). All the participants had English as their only transactional language. As expected, the HC group performed better on a modified digit-symbol substitution task (DSST) measuring processing speed and the Colour-Word Stroop task. Clinical and demographic characteristics are provided in Table 1. In the FES group, 50% of the sample were fully antipsychotic naïve while the other 50% were exposed to a mean of 2.8 days of a lifetime daily dose to antipsychotics. Of those in the FES sample exposed to antipsychotics, 50% were on antipsychotics with low dopamine occupancy and the other 50% were on antipsychotics with high dopamine occupancy (as defined by de Boer and colleagues^55^).

**Table 1:** Clinical and demographic characteristics of the sample at baseline.

	HC	FES	BF_10_	Effect size
	Mean ± SD	Mean ± SD		δ 95% CI
Age	21.4 ± 3.2	22.0 ± 3.6	0.308	−0.56, 0.24
Gender	67% male	77% male	0.509	−1.48, 0.46
Educational level (<12/>12 years)	27%/73%	37%/63%	0.474	−1.41, 0.46
PANSS-8 Positive	–	12.1 ± 3.0	–	–
PANSS-8 Negative	–	7.4 ± 4.3	–	–
PANSS-8 total	–	25.6 ± 6.8	–	–
SOFAS	80.2 ± 10	39.3 ± 13.3	>10000	
Parental SES ( < 3/ >3)	42% / 58%	33% / 67%	0.387	−0.55, 1.34
CDS	–	3.5 ± 3.3	–	–
CGI	–	5.2 ± 0.9	–	–
TLI total	0.28 ± 0.3	1.60 ± 1.3	>10000	−1.65, −0.69
TLI Disorganization of Thinking	0.153 ± 0.2	1.01 ± 1.1	674	−1.38, −0.45
TLI Impoverishment of Thinking	0.13 ± 0.2	0.58 ± 0.7	41.4	−1.17, −0.27
TLI Dysregulation	0.06 ± 0.16	0.17 ± 0.29	1.69	−0.85, −0.00
DSST	68.6 ± 11.3	52.8 ± 13.9	>10000	0.66, 1.63
Semantic Verbal Fluency	26.6 ± 6.9	19.8 ± 6.2	646	0.47, 1.45
Stroop total correct	78.2 ± 3.1	70.8 ± 13.1	19.93	0.22, 1.33
Stroop total time	74.6 ± 11.3	84.8 ± 17.0	11.12	−1.07, −0.17
Stroop IG	8.89 ± 1.5	7.09 ± 3.5	12.2	0.14, 1.02
Daily dose	–	0.81 ± 0.49		
Total dose		160.7 ± 110		

Mean and Standard deviations are shown for continuous variables, with percentages for categorical variables. BF_10_: Bayes Factor. *SOFAS* Social and Occupational Functioning Assessment Scale, SES: Parental socioeconomic status score. *CDS* Calgary Depression Scale, CGI-S Clinical Global Impressions Scale Severity of Illness, *TLI* Thought and Language Index, Impoverishment: Poverty of Speech + Weakening of Goal; Disorganized Thinking: Peculiar words + sentences + illogicality; Dysregulation: Perseveration + Distractibility. *DSST* Modified Digit Symbol Substitution Test. Stroop IG: Stroop interference score - Golden method. Daily dose: average Daily Defined Dose, Total Dose: total exposure calculated based on Daily Dose and number of days of exposure.

### Baseline differences in word similarity

**Fig. 1:**
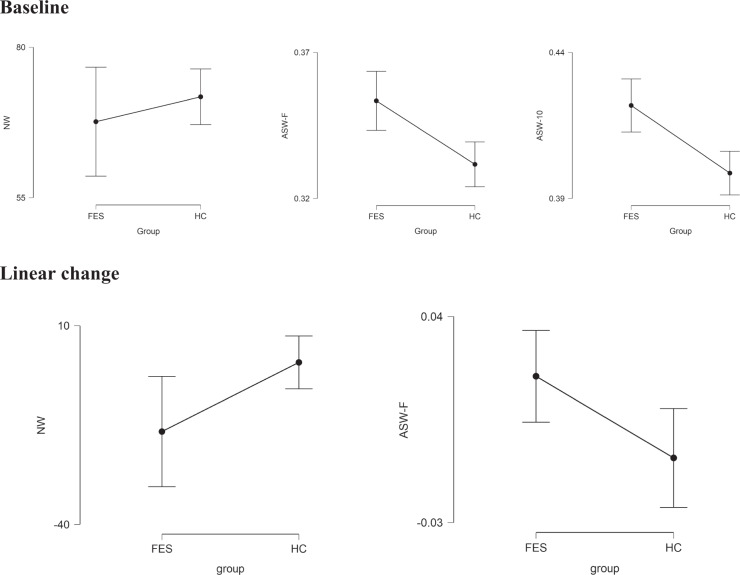
Group differences in linguistic variables at baseline and the change over time of linguistic variables. Descriptive plots of 95% credible interval between groups. NW Number of words, ASW-F Average Similarity of Words in Full picture description, ASW-10 Average Similarity of Words over moving window of 10 words, FES First Episode Schizophrenia, HC Healthy control.

In the description task, the groups did not differ in the number of words spoken but FES had higher similarity (ASW-F, BF_10_ = 6.53; ASW-10, BF_10_ = 32.76) compared to the HC group. These results are shown in Table 2 and Fig. 1. The increase in semantic similarity was specific to the picture description task; when we studied similarity of word choices in a category fluency task in a subsample of subjects (HC *n* = 33, FES *n* = 39), there was no difference among groups (ASW-F, HC: 0.497 ± 0.04; FES: 0.477 ± 0.05, BF_10_ = 0.696), indicating discourse-related specificity of increased semantic similarity in schizophrenia.

**Table 2:** Summary group differences at baseline.

	HC Mean ±SD	FES Mean ±SD	BF_10_	Effect size δ 95% CI
Number of words	70.6 ± 14.9	68.4 ± 30.3	0.249	−0.32, 0.48
ASW-F	0.334 ± 0.025	0.352 ± 0.034	6.53	−1.05, −0.17
ASW-10	0.400 ± 0.023	0.421 ± 0.031	32.76	−1.14, −0.25

*ASW-F* Average similarity of words – full picture description, *ASW-10* Average similarity of words – 10 words moving window. Note that the variables reported here are individually averaged across 3 speech samples per subject. BF_10_: Bayes Factor (alternate vs. null hypothesis).

### Longitudinal changes in word similarity

In the 6-month follow-up sample (*n* = 33, FES = 20, HC = 13), the 2 groups were matched for age (FES: 22.5 ± 5.0; HC: 21.5 ± 3.1, BF_10_ = 0.390) and gender (FES: 80% male; HC: 70% male, BF_10_ = 0.611). The follow-up sample of patients had no more than anecdotal evidence of differences at baseline with the dropped-out sample (PANSS BF_10_ = 0.302; TLI BF_10_ = 0.327; DSST BF_10_ = 1.699; ASW-F BF_10_ = 1.718). Patients with FES had strong evidence for functional improvement based on SOFAS scores (Baseline: 41.5 ± 13.5; follow-up: 61.0 ± 12.9; mean change = 19.5 ± 14.3; paired t test BF_10_ = 4868), and clinical improvement based on a reduction in PANSS-8 total score (Baseline: 25.2 ± 5.7; Follow-up: 15.1 ± 5.0, mean change = -10.25 ± 4.9; paired t test BF_10_ > 10000) from baseline to follow-up assessment, as expected following clinical intervention (medication doses detailed below). While average positive symptom scores improved (Baseline: 12.5 ± 2.6; Follow-up: 5.2 ± 1.7, BF_10_: > 10000), the average negative symptom scores of the PANSS did not show a notable change between baseline and the follow-up (Baseline: 6.8 ± 3.7; Follow-up: 7.1 ± 4.1, BF_10_: 0.255), indicating the persistent nature of this core feature of schizophrenia.

To study the longitudinal trajectory of word usage during descriptive discourse, we performed a Bayesian paired t-test from baseline to 6-month follow up in both groups. As shown in Table 3, the null model was more likely than the difference-between-measures model for the HC group across all linguistic variables, indicating relative stability of semantic similarity and the number of produced words among healthy subjects, when the same pictures were described twice in a period of ~6 months. In the FES group, the most notable difference between measures was seen in semantic similarity which was estimated from the full 1-min picture description (ASW-F; BF_10_ = 6.3; see Fig. 1). We did not see the same level of evidence for linear change in ASW-10 or the number of words. For further correlational analysis with cognitive and symptom factors, we selected ASW-F as the linguistic measure of interest.

**Table 3:** Summary of baseline and follow-up 6 months comparison.

	HC					FES					Comparison
	Baseline	6 months	Paired BF_10_	Linear change		Baseline	6 months	Paired BF_10_	Linear change		BF_10_ linear change *groups
	Mean ± SD	Mean ± SD		Mean ± SD	δ 95% CI	Mean ± SD	Mean ±SD		Mean ±SD	δ 95% CI	
Number of words	69.2 ± 13.9	70.0 ± 12.4	0.28	0.76 ± 11.0	−5.88, 7.41	66.9 ± 30.0	52.1 ± 19.8	1.74	−16.62 ± 29.6	−30.50, −2.750	0.13
ASW-F	0.332 ± 0.02	0.324 ± 0.01	0.44	−0.008 ± 0.028	−0.025, 0.009	0.337 ± 0.02	0.353 ± 0.03	2.07	0.020 ± 0.033	0.004, 0.035	6.32
ASW-10	0.398 ± 0.02	0.391 ± 0.02	0.38	−0.007 ± 0.028	−0.024, 0.010	0.407 ± 0.02	0.414 ± 0.02	0.61	0.011 ± 0.026	−0.001, 0.023	2.37

*NW* Number of words, *ASW-F* Average Similarity of Words in Full picture description, *ASW-10* Average Similarity of Words over moving window of 10 words. BF_10_: Bayes Factor. δ 95% *CI* Effect Size 95% credible interval.

### Symptoms, functioning, and word similarity

**Fig. 2:**
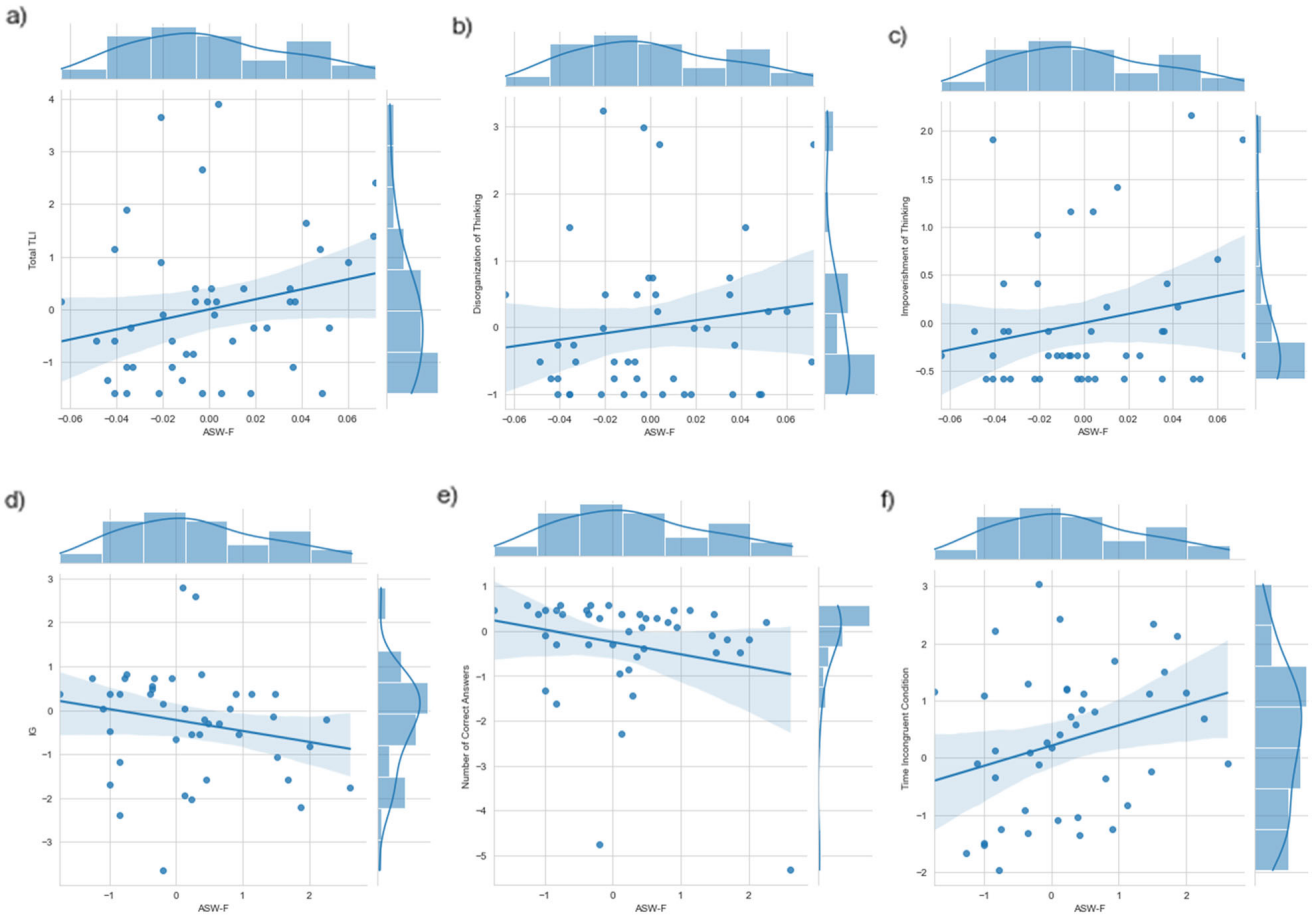
Correlation between ASW-F, TLI symptoms and Stroop scores in the patient group at baseline. ASW-F Average Similarity of Words in Full picture description with TLI (Thought Language Index) scores **a**) Total, **b**) Disorganization of thinking subscore and **c**) Impoverishment of thinking subscore; and with Stroop **d**) IG: Interference score, **e**) Number of correct answers and **f**) Response time incongruent condition.

Among FES subjects, ASW-F at the time of illness onset was higher in the presence of more severe positive symptoms (PANSS-8 positive r: 0.39, BF_10_: 9.24) and reduced functioning (SOFAS scores r: −0.41, BF _10_: 128) but this relationship was not seen with PANSS-8 negative (r: 0.08, BF_10_: 0.18) scores, TLI impoverishment (r: 0.21, BF_10_: 0.49), disorganization (r: 0.14, BF_10_: 0.28) or dysregulation (r: −0.06 BF_10_: 0.20) scores (Fig. 2). Among FES subjects that were followed-up, there was moderate evidence for increasing ASW-F in patients with increasing PANSS-8 negative (r: 0.592, BF_10_: 18.7) but not with change in PANSS-8 positive (r: −0.125 BF_10_: 0.435), or SOFAS scores (r: −0.04 BF_10_: 0.322).

### Cognition and word similarity

When all subjects (patients and controls) at the baseline were considered together, ASW-F was higher in subjects with reduced Stroop accuracy (r: −0.31, BF_10_: 13.3). The within-group effects were weaker, but in the same direction (FES only: r: −0.22, BF_10_: 1.01; HC only: r: −0.29, BF_10_: 1.61). Higher ASW-F scores also related to a lower Stroop Interference score (of Golden: IG) (r: −0.29, BF_10_ of 8.24,; FES only: r: −0.20, BF_10_: 0.81; HC only: r: −0.25, BF_10_: 1.13) and prolonged reaction time for the Stroop incongruent condition (r: 0.29, BF_10_: 8.6; FES only: r: 0.28, BF_10_: 1.97; HC only: r: 0.06, BF_10_: 0.29). This indicates that semantic co-occurrence in discourse production was higher in the presence of a cognitive control deficit indexed by reduced inhibitory control (poor accuracy) and information processing speed. A more specific index of serial processing speed derived from a modified Digit Symbol Substitution Test was also lower in the presence of increased ASW-F across the entire sample (r: −0.48, BF_10_: 304). This association was largely driven by the FES group (r: −0.41, BF_10_: 7.99), and not the HC (r: −0.03, BF_10_: 0.21) (see more details in the supplementary materials).

### Effects of antipsychotics exposure

We did not observe differences in ASW-F between the antipsychotic naïve, or low and high D2 receptor occupancy medication sub-samples at baseline (ANOVA, BF_10_ = 0.239), or between patients taking low and high occupancy drugs by the time of follow-up (t-test, BF_10_ = 0.607). To investigate possible dose effects of antipsychotics, we related both the Daily dose (average Daily Defined Dose) and Total Dose (total exposure calculated based on Daily Dose and number of days of exposure) to number of words and ASW-F at both time points. As shown in Table 4, the difference between the baseline and follow-up measures on number of words and ASW-F were not correlated with Daily Dose or Total Dose.

**Table 4:** Relationship between 6-months change in linguistic variables and medication dose.

	Pearson’s r	BF_10_	Lower 95% CI	Upper 95% CI
Daily Dose - NW	0.105	0.303	−0.330	0.491
Daily Dose- ASW-F	−0.161	0.343	−0.530	0.283
Total Dose - NW	0.083	0.293	−0.348	0.475
Total Dose- ASW-F	−0.225	0.424	−0.574	0.227

Daily dose = average Daily Defined Dose, Total Dose: total exposure calculated based on Daily Dose and number of days of exposure. *NW* Number of words, *ASW-F* Average Similarity of Words in Full picture description, *CI* credible intervals.

### Effect of social factors on word similarity

To investigate possible effects of immigrant status and the use of a language other than English at home^56^, we removed 20% of subjects that satisfied this criterion and analyzed the difference in ASW-F at baseline. We continued to see evidence in favour of increased semantic similarity among patients with FES (ASW-F BF_10_ = 6.46). Similarly, when patients were stratified according to education status (<12/>12 years) and by parental socioeconomic status (higher than median vs. lower than median) and were compared with each other, there was no difference in ASW-F or number of words (Educational background: ASW-F BF_10_ = 0.594; number of words BF_10_ = 0.173; Socio-economic status: ASW-F BF_10_ = 0.194; number of words BF_10_ = 0.148). These results indicate that word similarity is affected by the diagnosis of schizophrenia, rather than social factors that are often associated with the diagnosis.

## Discussion

Using a computational semantics approach, we examined word similarity during a controlled descriptive discourse task in untreated first-episode schizophrenia at baseline and after 6 months of treatment. We report four major findings. First, when faced with the task of describing an unfamiliar concrete referent (a picture), patients with schizophrenia choose words with a higher probability of semantic co-occurrence. The likelihood of this phenomenon is more pronounced when psychotic symptoms are severe and functional deficits are profound. Interestingly, this objectively verifiable linguistic feature of higher similarity is seen irrespective of the degree of clinically detectable thought disorder. Second, higher word similarity during the discourse was related to lower cognitive control (in the whole sample), as indexed by the Stroop task, and reduced processing speed (especially in patients), indicating a role for domain-general processes in aberrant word choices in schizophrenia. Third, the higher semantic similarity in patients was only present in the discursive task and not in the verbal fluency task. Four, despite symptomatic improvement with treatment (i.e., reduction of positive symptoms), the aberrant semantic similarity persisted with time, worsening especially in those with increasing burden of negative symptoms, but this was not explained by exposure to antipsychotics. Taken together, restricted sampling from the putative semantic space during a discursive discourse is likely to be a specific, persistent deficit in early stages of schizophrenia that follows the trajectory of negative symptoms.

Semantic impairment in people with schizophrenia is widely reported^61^, however, this evidence relies mostly on comprehension based experimental paradigms^62–64^ or experiments where the semantic retrieval demand, or route in the semantic space, is set by the researchers (stimulus with prime and target) and not chosen by the participants. Studies of the latter type generally involve category fluency tests, wherein patients have either no reduction in overall word similarity or choose adjacent words that are less similar^4,65^. In contrast to verbal fluency tasks, in a discursive task, there is a necessity to ‘forage’ widely to accomplish the goal of description. Such wide foraging appears to be diminished in schizophrenia^66^. We also note that such a narrowing of semantic sampling space relates to a higher Stroop interference effect; thus, a failure of the prefrontal executive control, either in a general- or domain-specific manner^67^, may influence word choice. The lack of control in the selection of the lexical items may lead to a restricted repertoire wherein a word and its activated associates^68,69^ dominate the unfolding discourse.

Contrary to our expectation, we did not find a relationship between semantic similarity and severity of formal thought disorder in this sample of FES. In general, the degree of shared variance between computational linguistic measures and observer-rated formal thought disorder scores have not been consistent^29,42,52,70–72^. In particular, while some sentence level structural measures (e.g., connective use^70^, narrativity and referential cohesion^73^) relate to thought disorder, the overall shared variance is small for word-level measures^73^. This also supports the view that semantic similarity (i.e., the distance among words inferred from distributional semantics) is a latent variable; pathological changes in semantic similarity are not immediately discernible in a clinical interview, even when qualitative word peculiarities are sought from transcribed speech. Nevertheless, greater variance in clinical ratings may be required to conclusively study this issue^44^.

Our study has several strengths as well as certain limitations. To our knowledge, this is the first longitudinal report on the nature of word choices made during a controlled discourse in patients with psychosis. Although the evolution of lexical and semantic deficits in schizophrenia is still not fully understood, meta-analytical evidence indicates no temporal change when category fluency is tested -indicating its fixed, endophenotype-like stability over time^74^. In contrast, we report that discourse-specific word choice deteriorates over time in the early stages of schizophrenia. Secondly, we estimated antipsychotic exposure meticulously over the follow-up period. The discourse-related word similarity did not change in proportion to antipsychotic dose exposure, in contrast with the reported influence of antipsychotic dose on other NLP measures such as syntactic complexity and percentage of time speaking^55^. We were limited in terms of the number of healthy controls for whom we had follow-up assessment of word similarity; nevertheless, this did not diminish our ability to demonstrate group differences in the longitudinal change scores based on within-subject variance. Secondly, our descriptive discourse was constrained by time; we do not know if the choice of words would have been less similar if the discourse was unconstrained and spontaneous. This needs to be examined in future studies with speech elicited in different contexts. Lastly, our sample of first-episode schizophrenia did not include the most unwell patients (not referred by clinicians) and those who were involuntarily hospitalised (deemed to lack capacity to consent) and drop-outs were substantial. While the patients who were unavailable for follow-up had a similar profile to those who were retained, we cannot rule out the possibility that they had better outcomes; we urge caution in generalising our results to this group.

In conclusion, we demonstrate that descriptive discourse in first episode of schizophrenia is characterized by an aberrantly high semantic co-occurrence that relates to functional deficits at initial presentation and persists despite treatment in the early stages. Given its relevance to social functioning, and our ability to measure it objectively in a non-invasive, repeated manner, we propose this measure to be a suitable computational linguistic measure that indexes one aspect of the hitherto unclear but persistent pathophysiology of schizophrenia.

## Methods

### Participants

Eighty-two English-speaking participants were recruited, including 46 experiencing their First Episode of Schizophrenia (FES) and 36 healthy controls (HC). FES participants were enrolled through the Prevention and Early Intervention for Psychosis Program of London Health Sciences Centre (London, Ontario, Canada) and were diagnosed with Schizophrenia according to the DSM-5 criteria, using the consensus procedure described by Leckman and the Structured Clinical Interview for DSM-5 to confirm diagnosis 6 months after the first presentation^75^. The severity of symptoms was confirmed with the Positive and Negative Syndrome Scale-8 items version (PANSS)^76^. The FES participants were in the acute phase of the illness and drug-naïve for antipsychotics at the time of the first assessment with a maximum equal to or less than 14 days of total lifetime antipsychotic use. We used a consecutive referral strategy for patient recruitment whereby all patients referred to the only first episode clinic in the catchment area between April 2017 and June 2019 were approached, if deemed to have the capacity to consent for the study by the clinicians.

We also recruited a HC group from the same geographical catchment as patients, through pamphlets and word-of-mouth advertisement. Healthy subjects were group-matched with FES for age, sex, level of completed formal education and parental socio-economic status. The inclusion criteria for HC group included no personal or family mental illness or neurological diseases, prior or current antipsychotic exposure, active substance dependence or the inability to provide informed consent.

All participants provided written informed consent before assessment and ethics approval was granted by the Human Research Ethics Board at Western University, London, Ontario.

Thirty-three participants, 20 with schizophrenia (SZ) and 13 HC, were followed up approximately 6 months from the first assessment (x̄ = 214.9 ± 44.9 days). The medication exposure of the FES group was calculated according to the Daily Defined Dose (DDD) methodology^77^, and D2-occupancy based classification followed the description of de Boer and colleagues^55^. To calculate total exposure, we considered the type of medication, the dose prescribed, the number of days of effective exposure based on treatment compliance over the follow-up time measured using an established instrument^78^ for adherence that correlates well with pill counts^79^. As reported in our prior study^80^, nearly 50% of patients went on long-acting injection by the 1^st^ month of treatment, further ensuring treatment compliance.

### Assessments

All participants were assessed with the Social and Occupational Functioning Assessment Scale (SOFAS) to quantify the level of functioning in social and occupational domains, without overlapping with symptom measurements^81^ and with the Socioeconomic Status (SES) to measure the parental level of occupation and employment from 1 (Managerial and professional occupations) to 5 (routine occupations)^82^. The FES group was assessed with the Calgary Depression Scale (CDS)^83^ covering depressive symptoms over the past 2 weeks and with the Clinical Global Impression Scale Severity of Illness (CGI-S) to assess the overall severity from 1 (normal) to 7 (among the most extremely ill patients)^84^.

Participants were assessed using a modified version of the digit symbol substitution task (oral and written version) used in our previous studies^22,85,86^, semantic verbal fluency task in its original version and the Colour-Word Stroop test in an adapted version used in other studies^87,88^. The DSST oral and written versions were scored by counting the number of correct symbols within the allowed time, with the total DSST score being calculated by averaging the oral and written version scores. For the fluency task, participants were instructed to generate as many words as possible within one minute from the semantic category of animals, and the metric of average similarity across the full set of response was measured using CoVec (see next section). In the Stroop test, the performance was measured by the number of correct answers, the response time in incongruent conditions and the Interference score (IG). The IG was computed with Golden method^89^, in which we calculated the number of correctly named items in each condition: Word score = number of words read correctly, Colour score = number of colour hues named correctly, and Colour-Word score = number of colour hues named correctly. Then we estimated the Predicted Colour-Word score with the product of the Word and Colour scores with the following formula: Predicted Colour-Word score = (Word score x Colour score) / (Word score + Colour score). Finally, the interference score (IG) was computed subtracting the Predicted Colour-Word score from the actual number of correctly named items in the Colour-Word incongruous condition^90^.

The discourse task was the description of 3 images and the scoring was done using the Thought Language Index (TLI). The TLI is a reliable instrument to assess formal thought disorders under standardized conditions^3^. The participants were asked to describe Thematic Apperception Test^91^ images and were given one minute for each image. The interviewer prompted the participants to continue if they stopped speaking before the stipulated time. The interview was recorded and later transcribed by research assistants. The transcriptions were then analyzed with the Covington Vector semantic tool^92^.

### Semantic Analysis

The Covington Vector semantic tool (CoVec) is a natural language processing tool based on data from the Global Vectors for Word Representation (GloVe) Project, with 840 billion words in English on a 300-element vectors^93^. GloVe measures the likelihood of co-occurrence of words through vector cosine similarity based on overall statistics of how often the word appears given the context (P(w | c)). The GloVe project is a count-based model with a large matrix of (words*context) co-occurrence information that is normalized by log-smoothing the matrix. Covec reports the average of similarity, that is, whether successive words are commonly used in the same context (or together), with an n-word frame segment, using all the positions of the frame. Before processing the text, CoVec removes punctuation, marks ‘stop words’ (eg. “a”, “the”, “is”, “at”, among others), and finally, ignores words that are not found in the GloVe dataset (displays a warning of all the missing words). The metrics used include the Number of words (NW), average similarity of words in the full-frame of the text (ASW-F) or in 10 words moving window (ASW-10). Note that ASW is described as Coherence in CoVec’s output.

### Data analysis

Clinical and demographic data were analyzed using descriptive and Bayesian statistics (Bayesian t-test for continuous variables and Bayesian Chi-square between categorical variables). We first compared group performance with a Bayesian t-test on the number of words and semantic similarity variables. To compare the progression of language features, we conducted a Bayesian paired t-test between baseline and 6-month follow-up measures, then, we estimated the linear change between measures and compared it between groups. We used a Bayesian ANOVA to explore the differences between the types of medication in the FES sample. We conducted a Bayesian Pearson correlation to explore the effect of antipsychotics on our language variables. To address the interaction with cognitive and symptom variables, a Bayesian correlation was conducted between semantic co-occurrence and Stroop, DSST, TLI and PANSS scores. The variables were correlated considering the linear change between baseline and follow-up and were standardized by dividing the linear change with the baseline. Finally, we tested the effect of the use of a language other than English, educational background and socio-economic status of the parents with Bayesian t-test for two groups stratification and Bayesian ANOVA for three groups stratification. The prior distribution for the parameter was set by default and all reported statistical tests were two-sided; no transformations were undertaken on any data. Effect sizes are presented as correlation coefficients [r] or Cohen’s delta [δ], with 95% credible intervals reported for both measures. All the statistical analyses used JASP version 0.14.0.1^94^ and the figures were made on Python in Jupyter Notebook 6.1.5^95^.

## Supplementary information

Correlation between linguistic and cognitive measures

## Data Availability

The transcripts used for this study are currently prepared to be archived at talkbank.org. These transcripts, as well as anonymised clinical scores are available from the corresponding author upon reasonable request within the stipulations laid by The Research Ethics Committee of University of Western Ontario, London, Canada.

## Code availability

The codes for generating figures (Jupyter) are available upon request.
