# Supplementary material for: Progressive changes in descriptive discourse in First Episode Schizophrenia: a longitudinal computational semantics study
Source: Schizophrenia (Heidelb). 2022 Apr 12;8(1):36. doi: 10.1038/s41537-022-00246-8 (PMC9261094; doi:10.1038/s41537-022-00246-8)

Supplementary materials

Correlation between ASW-F and Stroop ACC

FES HC All


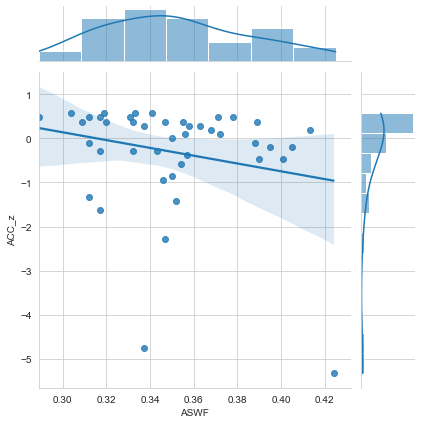

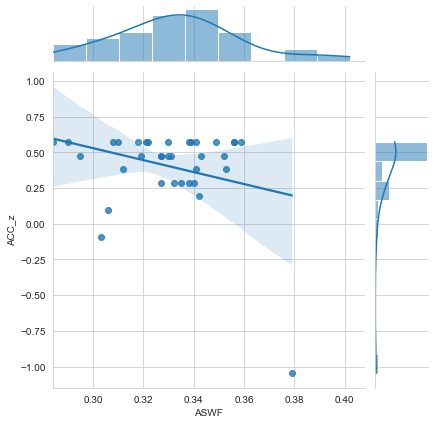

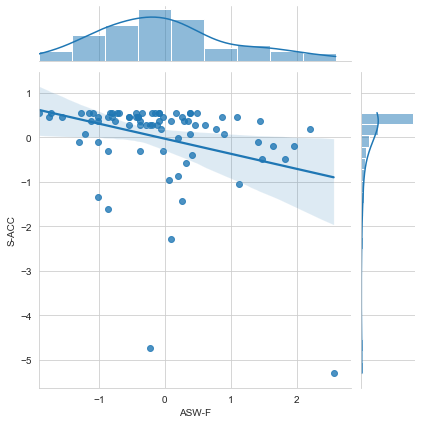


Correlation between ASW-F and Stroop IG

FES HC ALL


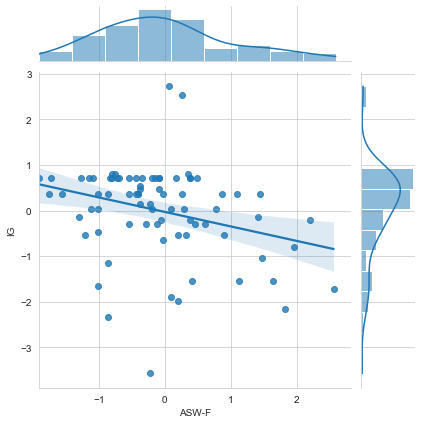

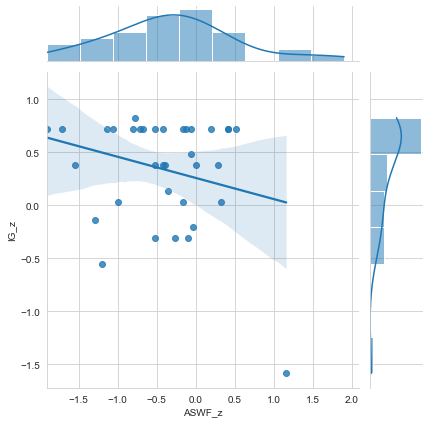

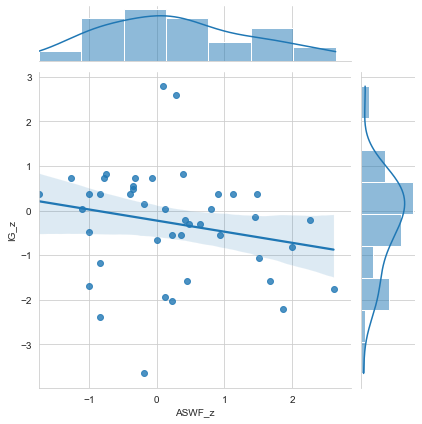


Correlation between ASW-F and Stroop total time incongruent condition

FES HC All


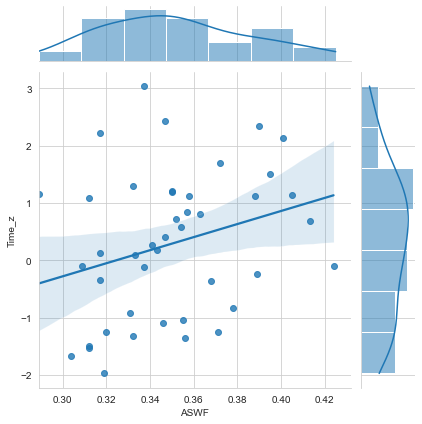

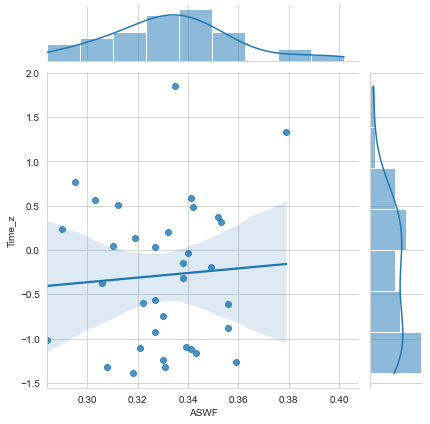

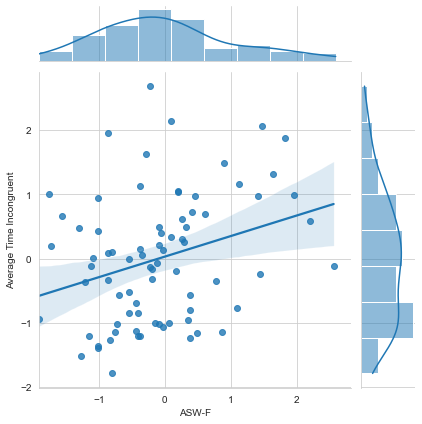


Correlation between ASW-F and DSST

FES HC All


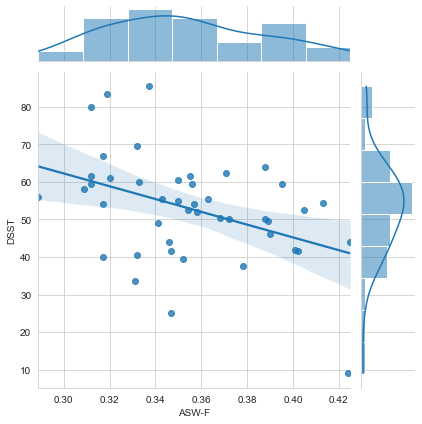

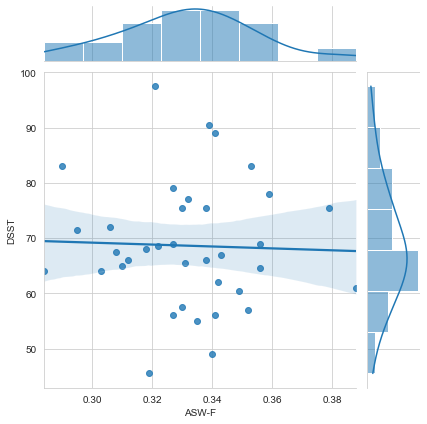

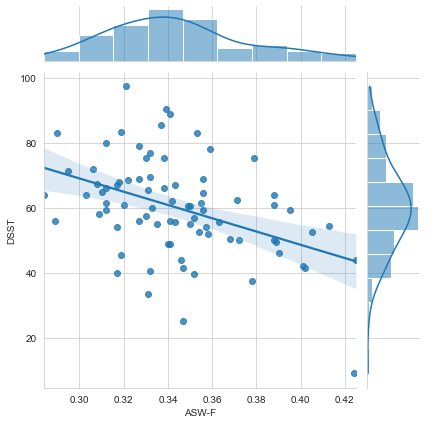


Correlation between ASW-F and DSSTw

FES HC All


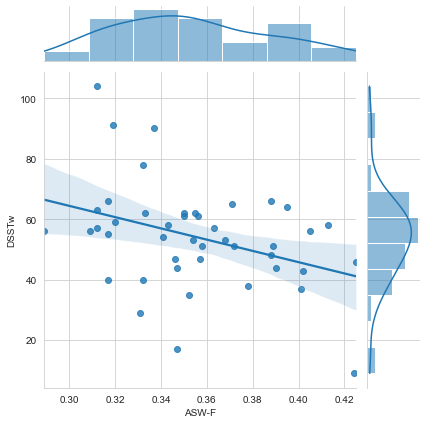

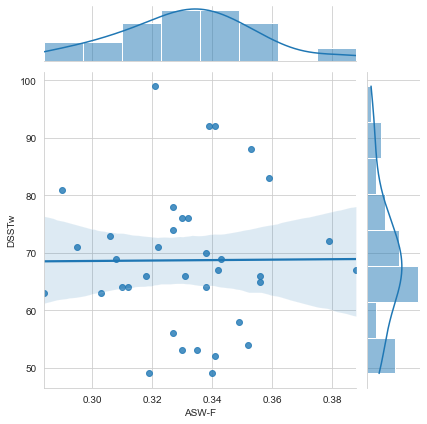

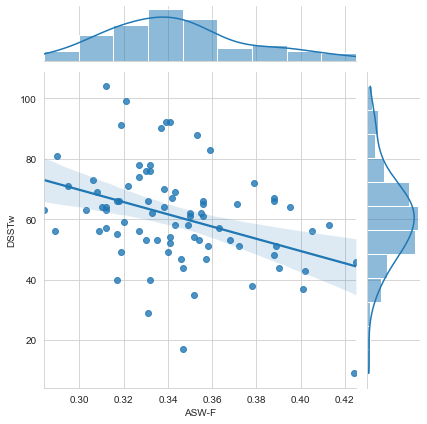


Correlation between ASW-F and DSSTo

FES HC All


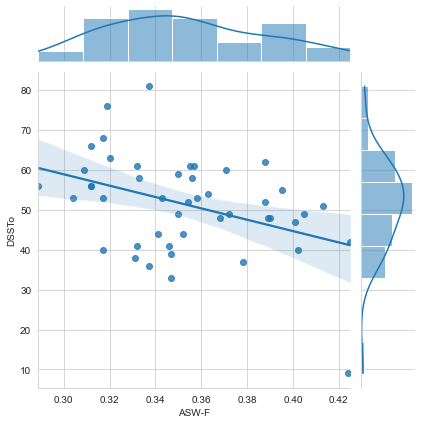

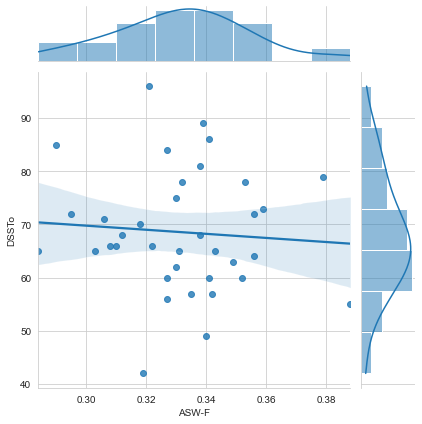

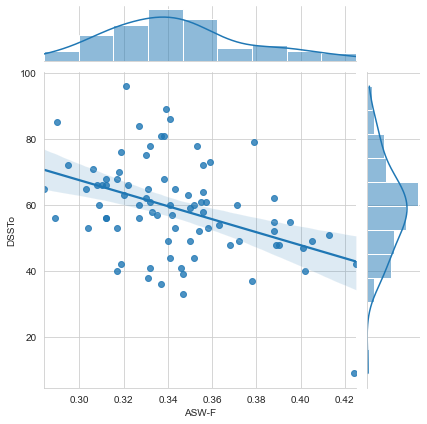

Supplement: Supplementary file 1 — Correlation between linguistic and cognitive measures [file 41537_2022_246_MOESM1_ESM.docx]
